# *Verbena officinalis* L. Herb Extract, Its Amino Acid Preparations and 3D-Printed Dosage Forms: Phytochemical, Technological and Pharmacological Research

**DOI:** 10.3390/plants14172651

**Published:** 2025-08-26

**Authors:** Oleh Koshovyi, Getter Dolgošev, Udhan Wimukthi Meegama, Koit Herodes, Yurii Hrytsyk, Lyubov Grytsyk, Andriy Grytsyk, Igor Kireyev, Jyrki Heinämäki, Ain Raal

**Affiliations:** 1 Institute of Pharmacy, Faculty of Medicine, University of Tartu, Nooruse 1, 50411 Tartu, Estonia; oleh.koshovyi@ut.ee (O.K.); getter.dolgosev@ut.ee (G.D.); jyrki.heinamaki@ut.ee (J.H.); 2 Institute of Chemistry, Faculty of Science and Technology, University of Tartu, 50411 Tartu, Estonia; udhan.wimukthi.meegama@ut.ee (U.W.M.); koit.herodes@ut.ee (K.H.); 3 Department of Pharmaceutical Management, Drug Technology and Pharmacognosy, Ivano-Frankivsk National Medical University, 76018 Ivano-Frankivsk, Ukraine; ygritsik@gmail.com (Y.H.); grycyk_l@ukr.net (L.G.); grycyk@ukr.net (A.G.); 4 Institute for Advanced Training of Pharmacy Specialists, The National University of Pharmacy, 61002 Kharkiv, Ukraine; ivkireev@ukr.net

**Keywords:** *Verbena officinalis* L., herb extract, phenolic compounds, amino acids, neurotropic activity, 3D printing

## Abstract

Vervain (*Verbena officinalis* L., Verbenaceae family) is a perennial plant which grows widely in Europe. It is rich in iridoids, phenolic acids, phenylpropanoid glycosides, flavonoids and terpenoids. Verbena has traditionally been used in folk medicine to calm the nervous system, but there is a lack of scientific data about it. The aim of this study was to explore and characterise the chemical profile and neurotropic effects of *V. officinalis* dry extracts and their amino acid-based preparations. We determined a total of eight main phenolic compounds and 17 amino acids in the *V. officinalis* dry extracts. To evaluate the neurotropic effects of the verbena extracts, the following behavioural pharmacology tests were used: Open Field Test, Elevated Plus Maze, Black-and-White Box Test and Tail Suspension Test. The dry aqueous–ethanolic extract (extractant 70% ethanol) demonstrated strong anxiolytic and antidepressant effects, while its dry modified extracts with valine and arginine consistently exhibited pronounced sedative activity across all studies. For example, the Tail Suspension Test demonstrated that the total immobility time in animals receiving the dry aqueous–ethanolic extract was the lowest, being 1.22-fold (*p* < 0.05) lower than in control animals and 2.25-fold (*p* < 0.05) lower than in the animals treated with the reference drug preparation (“Sedaphyton”). A novel aqueous-based gel formulation feasible for semi-solid extrusion (SSE) 3D printing was designed. This printing gel enables the fabrication of new oral dosage forms for *V. officinalis* dry extracts. The effects of pharmaceutical preparations on the human central nervous system require clinical studies.

## 1. Introduction

Vervain or common verbena (*Verbena officinalis* L., Verbenaceae) is a perennial plant that grows widely in Europe. The plant also spreads to North America, Asia and North Africa [1].

*V. officinalis* is rich in iridoids, phenolic acids, phenylpropanoid glycosides, flavonoids and terpenoids [2]. The most characteristic iridoids of this plant raw material are verbenaline and hastatoside. The iridoid glycosides, such as 3,4-dihydroverbenaline, aucubin, 7-hydroxydehydrohastatoside, and the secoiridoids, such as verbenoside A, verbeofflin I and verbenoside B, have also been identified [3,4,5]. The most prevalent phenylpropanoid glycosides of the plant include verbascoside, isoverbascoside and eukovoside, while isomers of leukoseptoside and cistanoside are also present [6]. Regarding fatty acids, the most abundant compounds are α-linolenic acid, palmitic acid, linoleic acid and oleic acid. The phenolic acids identified in the plant include gallic acid, syringic acid, ferulic acid, cinnamic acid and protocatechuic acid, along with quinic, chlorogenic, rosmarinic acids and dicaffeoylquinic acid derivatives [2,7]. Among the flavonoids found in *V. officinalis*, notable compounds include apigenin, luteolin, 5,7,4′-trihydroxy-8-methoxyflavone, scutellarein and its glucoside derivatives (scutellarein 7-glucoside, scutellarein-7-diglucuronide and scutellarein-7-glucuronide). Additionally, the plant contains pedalitin, pedalitin-6-galactoside, quercetin, kaempferol, isorhamnetin, diosmetin and rutin [2,8,9,10].

The essential oil composition of *V. officinalis* varies greatly depending on the geographical location and the specific plant part used in distillation. The plant contains mono-, di-, tri- and sesquiterpenoids [11,12]. Among monoterpenoids, the key compounds include citral, limonene, eucalyptol, menthol, α-pinene, β-pinene, sabinene and β-phellandrene. Carnosol and rosmanol are identified as the diterpenoids of the plant [13,14]. The most abundant sesquiterpenoids are caryophyllene oxide, α-curcumin, β-caryophyllene, hexahydrofarnesylacetone and spathulenol. The dominant triterpenoids include squalene, ursolic acid, barbinervic acid and oleanoic acid [2,15]. Regarding sterols, the plant yields β-sitosterol, γ-sitosterol, daucosterol, stigmasterol, campesterol and androst-5,15-dien-3-ol-acetate from its aerial parts [8,13,14,15].

Since 2008, a specific monograph on *V. officinalis* has been included in the European Pharmacopoeia (Ph. Eur.), and according to this monograph, the present plant must contain a minimum of 1.5% of verbenaline [16]. In addition to the abovementioned monograph, Ph. Eur. presents also another monograph for lemon verbena leaf (*Verbenae citriodorae folium*, *Verbena citriodora* (Palau) Cav.), also known as *Aloysia citriodora* Palau, with synonyms *Aloysia triphylla* (L’Her.) Kuntze, *Verbena triphylla* L’Her. and *Lippia citriodora* Kunth. The raw material is standardised based on its phenylethanoid content, specifically the tyrosol derivative acteoside (verbascoside), which must comprise a minimum of 2.5% of dry weight, expressed as ferulic acid. The essential oil content should be at least 3.0 mL/kg in the whole drug and 2.0 mL/kg in the fragmented drug (dry weight) [16].

Verbena has traditionally been used in folk medicine to support nervous system health and aid conditions, such as stress, anxiety, depression and insomnia. Moreover, it has been utilised for headache relief, premenstrual tension and the treatment of cramps, jaundice and asthma [17,18]. Externally, verbena herb infusion serves as a gargle for tonsillitis and stomatitis, and as a lotion for skin conditions. It is also incorporated in numerous herbal formulations, such as Sinupret^®^ (Bionorika, SE), which is a complex plant-based preparation known for its effectiveness in managing acute viral rhinosinusitis in children, thus helping to accelerate symptom relief [19].

The antioxidant, anti-inflammatory and hepatoprotective effects of *V. officinalis* have also been validated in separate studies [3,6,20,21]. Besides its antioxidant properties, common vervain leaf extracts have presented antimicrobial and antifungal activity. Moreover, the caffeoyl derivatives of such leaf extracts have shown a strong antifungal effect against *Penicillium expansum* and *Rhizopus stolonifera* [22].

The tumour growth-inhibitory properties of common vervain aqueous extract have been evaluated in vitro on rat and human colon adenocarcinoma cell lines. These studies revealed that polysaccharides from *V. officinalis* effectively suppressed colorectal cancer cell invasion and metastasis [23]. Moreover, two newly identified phenylethanoid glycosides exhibited cytotoxic activity comparable to vinblastine sulfate, a standard chemotherapy agent [24]. Methanolic extracts of semi-purified fractions demonstrated tumour growth inhibition across multiple melanoma cell lines [25]. Further animal studies confirmed its potential in hepatocellular carcinoma treatment. The administration of aqueous extract led to a 38.78% tumour size reduction compared to controls [26].

Gharachorloo and co-workers studied the antioxidant and antibacterial properties of the essential oil isolated from *V. officinalis* against *Staphylococcus aureus* and *Escherichia coli* [27]. The authors reported that *E. coli* exhibited greater susceptibility to yarrow essential oil compared to *S. aureus*, despite the fact that gram-negative bacteria generally display lower sensitivity to essential oils. The authors also found that increasing the essential oil concentration correlated with enhanced antioxidant activity [27]. According to a more recent study, silver nanoparticles synthesised from common vervain leaf extract demonstrated antibacterial effects against both gram-positive and gram-negative bacteria [28]. The potential of common vervain in managing hyperlipidaemia has also been explored. Crude plant extract was shown to decrease total cholesterol, triglycerides, low-density lipoproteins (LDL) and very low-density lipoproteins (VLDL) levels in vivo, and these results were comparable to the results obtained with atorvastatin as a reference drug [29]. It has been suggested that *V. officinalis* influences lipid metabolism, and quercetin, luteolin and kaempferol were identified as key bioactive compounds beneficial for atherosclerosis treatment [8]. The impact of common vervain aqueous extract on physical stress was further investigated in an animal study, and the present study revealed antioxidant modifications in red blood cell membranes and a significant protective effect against physical stress. Therefore, due to these beneficial properties, common vervain extract was suggested as a promising candidate for inclusion in sports supplements aimed at accelerating post-exercise recovery [30]. In addition, the biologically active compounds of *V. officinalis* have been demonstrated to have promising effects on depression, anxiety and insomnia [31,32,33].

Modifying biologically active substances found in plant extracts can significantly enhance their therapeutic potential. One widely adopted strategy involves the conjugation of extract constituents with amino acids [34,35,36]. For example, linking valine to acyclovir led to the formation of valacyclovir, a compound that substantially improves acyclovir’s systemic bioavailability, thereby increasing patient convenience and treatment efficacy [37]. It has also been shown that arginine enhances the absorption and stability of perindopril while simultaneously mitigating its adverse effects [38]. Modification of bioactive plant components or co-supplementation with an existing therapeutic defines a novel approach to address bioactivities or challenges with pharmacokinetics, showing the potential of innovative bio-based methods to solve limitations in drug development.

The incorporation of amino acids into an extract of *Leonurus cardiaca* L. resulted in novel preparations exhibiting stronger anxiolytic properties [39]. Moreover, combining arginine with extracts of highbush blueberry (*Vaccinium corymbosum* L., Ericaceae) [40] and cranberry leaves (*Vaccinium macrocarpon* Aiton, Ericaceae) [36] led to the development of new active substances with potential hypoglycaemic and lipid-lowering activities. These cases underline the potential of modifying *V. officinalis* herb extracts to generate new biologically active phytosubstances.

Herbal medicinal products are generally known for their favourable safety profiles. However, galenic formulations—such as tinctures, teas, decoctions and liquid extracts—often encounter limitations related to insufficient standardisation and reduced patient adherence. A novel, innovative approach to overcome these challenges is the application of pharmaceutical 3D-printing technologies [41,42,43], which allow the development of advanced oral dosage forms aimed at improving both the therapeutic efficiency and patient compliance of phytomedicines. Considering the broad range of *V. officinalis* extracts’ therapeutic applications, further studies are warranted to explore its phytochemical composition and pharmacodynamic properties, and to determine the most suitable pharmaceutical delivery systems for its extracts. The development of 3D-printed preparations containing *V. officinalis* extracts could offer a more precise, effective and patient-oriented approach to the use of herbal medicines in modern clinical and pharmaceutical settings.

The objective of this study was to explore and characterise the chemical profile and neurotropic effects of *V. officinalis* dry extract and its amino acid-based preparations. Additionally, we designed a novel aqueous gel formulation feasible for semi-solid extrusion (SSE) 3D printing, thus enabling the fabrication of new printed oral dosage forms loaded with *V. officinalis* dry extract.

## 2. Results

The *V. officinalis* extracts were yellow-brown-greenish powders with a characteristic smell. The yields of the dry extracts V_1_ (extractant 70% ethanol) and V_2_ (extractant water) were 13.62% and 18.44%, respectively.

### 2.1. Phytochemical Research

The phenolic compounds and amino acids in the *V. officinalis* extracts were studied by LC-MS (Table 1). A total of eight phenolic compounds and 17 amino acids were determined.

### 2.2. Pharmacological Research

The studied *V. officinalis* extracts (dissolved in water) were administered to animals orally as a single dose at 50 mg/kg. The control group animals received the “Sedaphyton” tablets at the same 50 mg/kg dose. Each tablet of “Sedaphyton” contains the following soft extracts as active ingredients: 0.05 g of valerian rhizomes with roots (*Valerianae radix cum radicibus*) in a 1:2.5 ratio, extracted with 40% ethanol; 0.03 g of motherwort herb (*Leonuri herba*) in a 1:3.0 ratio, extracted with 40% ethanol; and 0.03 g of hawthorn fruits (*Crataegi fructus*) in a 1:1.7 ratio, extracted with 70% ethanol. Control animals were given an equivalent volume of drinking water. For 3–4 h before administration, the animals were deprived of food but had free access to water. The study began 1 h after the administration of the extracts. The experimental results were processed using standard statistical methods, including the arithmetic mean, standard deviation, Student’s t-test and significance coefficient.

The “Open Field” test was based on assessing the natural defensive and exploratory behavioural reactions of rodents in a new open space [44,45,46]. The results of the study on the neurotropic activity of the *V. officinalis* extracts are presented in Table 2.

The results of the “Open Field” test (Table 2) showed that the animals administered V_1_-Val and V_1_-Arg extracts exhibited a 1.49-fold (*p* < 0.05) and 1.11-fold reduction in locomotor activity compared to control animals. In contrast, the animals receiving V_1_, V_1_-Gly, V_1_-Phe and V_1_-Lys extracts showed a slight increase in locomotor activity, but the difference was not statistically significant compared to control animals. After the administration of V_2_ extract, an increase in locomotor activity was observed. The locomotor activity was 1.25 to 1.28 times higher (*p* < 0.05) compared to control animals and a comparison group, respectively. The indicators of exploratory behaviour (number of vertical stands) were slightly higher with the animals administered V_1_ and V_2_ extracts compared to control animals. The administration of V_1_-Gly, V_1_-Phe and V_1_-Lys extracts had only a minimal impact on this indicator. The number of hole explorations was lower in all experimental groups compared to the intact animal group. The exploratory behaviour indicators in the animals receiving V_1_-Val and V_1_-Arg extracts were lower than in the comparison group treated with “Sedaphyton”. The number of vertical stands decreased by 1.17-fold and 1.80-fold (*p* < 0.05), respectively. Perhaps surprisingly, the number of hole explorations remained at the same level as was found with a comparison group. The verbena extracts did not significantly affect the emotional response indicators of mice in the “Open Field” test. Overall, the effects of V_1_-Val and V_1_-Arg extracts on the neurotropic activities in mice were slightly (1.02 to 1.21 times) higher than those observed in the comparison group receiving “Sedaphyton”. This was found when analysing the relative units of all neurotropic activities. Considering all types of activities in the “Open Field” test, it is evident that the administration of V_1_, V_2_ and V_1_-Gly extracts in mice resulted in a mild anxiolytic effect, and this effect exceeded the corresponding effect with control animals by 19.4%, 18.4% and 10.8%, respectively. The total activity indicators of V_1_-Phe and V_1_-Lys extracts were comparable to those of control animals, while the V_1_-Val and V_1_-Arg extracts showed similar effects to the reference drug “Sedaphyton”. This suggests a sedative effect of the modified verbena extracts after oral administration.

The Elevated Plus Maze test is another model for assessing situational anxiety, and this test allows the evaluation of the anxiolytic, psychostimulant and sedative effects of pharmacological agents [44,46]. This test is designed to study rodent behaviour under conditions of variable stressogenicity, where they can freely choose comfortable environments. It helps to assess anxiety levels, including exploration of dark compartments, natural fear of open spaces and height-related anxiety. The anxiolytic effect of a substance is evaluated based on the time spent in the dark and illuminated arms of the maze. Increased time spent in illuminated arms, along with more instances of peeking out and hanging from open sections, is classified as an anxiolytic effect. Overall motor activity reduction may indicate a sedative effect, while increased activity without changes in time spent in open arms suggests a psychostimulant or nonspecific motor influence. The test duration was 5 min, during which behavioural characteristics of mice were visually recorded. Such behavioural characteristics include the time spent in the open arm of the maze (exploratory activity), the time spent in the closed arm of the maze, the number of peeks from the closed arm, the number of downward glances from the ends of open arms (risk assessment), and the number of crossings over the central platform. The results of the study on the effects of the verbena extracts on mouse behaviour are presented in Table 3.

The results of the Elevated Plus Maze test (Table 3) showed that the administration of V_1_, V_1_-Phe and V_1_-Lys extracts led to significant changes in the emotional and behavioural responses of experimental animals in the test compared to the control animals and those receiving the comparison drug “Sedaphyton”. Regarding exploratory activity, the time spent in the open arm of the maze increased by 2.60-fold (*p* < 0.05), by 2.46-fold (*p* < 0.05) and by 1.95-fold (*p* < 0.05) after the administration of V_1_, V_1_-Phe and V_1_-Lys extracts, respectively (compared to control animals). Compared to the comparison group, the increase was 3.40-fold (*p* < 0.05), 3.21-fold (*p* < 0.05) and 2.55-fold (*p* < 0.05), respectively. The number of downward glances from the ends of open arms (risk assessment) significantly increased by 2.17-fold (*p* < 0.05) after the administration of V_1_, 2.42-fold (*p* < 0.05) after the administration of V_1_-Phe and 2.35-fold (*p* < 0.05) after the administration of V_1_-Lys compared to control animals. In addition, the animals receiving V_1_, V_1_-Phe and V_1_-Lys extracts showed an increase in crossings over the central platform, while the number of peeks from dark arms remained unchanged compared to control animals. After the administration of V_2_, V_1_-Gly and V_1_-Arg extracts, the time spent in open arms, the number of downward glances and the number of crossings over the central platform did not differ from those observed with the control animals. Conversely, the oral administration of V_1_-Val extract in mice appeared to induce a sedative effect. The time spent in the open arm of the maze significantly decreased by 2.54-fold (*p* < 0.05) compared to the control animals and 1.95-fold (*p* < 0.05) compared to the comparison group. A significant reduction in the number of downward glances was observed with the mice receiving V_1_-Val extract. The decrease in the number of downward glances was 4.67-fold (*p* < 0.05) compared to the control animals and 3.00-fold (*p* < 0.05) compared to the “Sedaphyton”-treated animals. Moreover, the number of peeks from the closed arm and crossings over the central platform decreased by 3.93-fold (*p* < 0.05) and 1.82-fold compared to the control animals. Therefore, our results suggest the presence of an anxiolytic effect in mice after the oral administration of V_1_, V_1_-Phe and V_1_-Lys extracts (Table 3). This was evidenced by increased exploratory and locomotor activity in mice. It is evident that V_1_, V_1_-Phe and V_1_-Lys extracts exert an activating effect on the CNS by reducing fear perception and enhancing adaptation speed to new conditions. The results for V_1_-Val suggest the presence of a sedative effect in mice.

The anxiolytic effect of the verbena herb extracts on the exploratory reactions of mice under stress conditions was studied using a “Black-and-White Box” test [44,45,47]. The animals were first placed in the dark compartment of the apparatus for 2 min to adapt to the dark environment. After this adaptation period, the experiment began, and for 3 min, the following parameters were recorded: time spent in the dark compartment (burrow reflex), number of peeks and exits into the illuminated compartment and the time spent in the illuminated compartment. It is known that anxiolytics increase the number of exits and the time spent in the illuminated compartment, whereas control animals prefer to remain in the dark compartment. The results of the study are presented in Table 4.

All three *V. officinalis* extracts showed anxiolytic activity in mice, and the most pronounced activity was observed with V_1_ extract (Table 4). After the administration of V_1_ extract, the mice showed an increase in time spent in the illuminated compartment. This increase in time was 1.28-fold (*p* < 0.05) higher compared to control animals and 1.68-fold (*p* < 0.05) compared to the animals receiving “Sedaphyton”. High anxiolytic activity was also observed with the V_1_-Gly and V_1_-Phe extracts. After the administration of these two extracts, the time spent in the illuminated compartment increased by 1.31-fold (*p* < 0.05) for V_1_-Gly and by 1.35-fold (*p* < 0.05) for V_1_-Phe compared to control animals. Compared to “Sedaphyton”-treated animals, the time spent in the illuminated compartment increased by 1.73-fold (*p* < 0.05) and 1.77-fold (*p* < 0.05) after the administration of V_1_-Gly and V_1_-Phe, respectively. Following the administration of V_1_-Arg, a decrease in exploratory behaviour was observed in mice, and the time spent in the illuminated compartment decreased by 1.46-fold (*p* < 0.05) compared to control animals. With the mice, the time spent in the illuminated compartment was at the same level as was found with the “Sedaphyton”-treated group. These findings suggest a predominantly sedative effect of V_1_-Arg extract. After the oral administration of V_1_-Lys and V_1_-Val extracts to the mice, no statistically significant anxiolytic effect was observed compared to control animals. We used the values for the time spent in the illuminated compartment and the number of exits from the dark section to calculate the average time of a single stay in the illuminated compartment (Table 4). The highest value for the average time of a single stay in the illuminated compartment was found after the administration of V_1_ extract to the mice (47.98 ± 12.85 s), while the lowest value was observed with the mice after the administration of V_1_-Arg extract (11.66 ± 2.66 s). Therefore, it is evident that the V_1_, V_1_-Gly and V_1_-Phe extracts exhibit anxiolytic effects, while V_1_-Arg extract presents a sedative effect.

The Tail Suspension Test was used for the initial assessment of the antidepressant activity of the verbena extracts [44,48,49]. This test models the animal behaviour based on despair. The Tail Suspension Test provides a quick and reliable assessment of antidepressant behaviour in mice. For 6 min, the behaviour of experimental animals was observed, and the immobility time (motionless hanging) was recorded. The results of the study are presented in Table 5.

The Tail Suspension Test (Table 5) showed that the total immobility time in mice receiving V_1_ extract was the lowest (84.67 ± 5.38 s), and this value was 1.22-fold (*p* < 0.05) lower than the value observed with control animals and 2.25-fold (*p* < 0.05) lower than the value recorded with the animals treated with “Sedaphyton”. Following the administration of V_1_-Phe and V_1_-Lys extracts, the total immobility time of mice decreased by 1.96-fold (*p* < 0.05) and 1.91-fold (*p* < 0.05) compared to the “Sedaphyton”-treated group. However, the difference in the total immobility time was not statistically significant compared to the value obtained with the intact mice. After the administration of V_2_, V_1_-Gly, V_1_-Val and V_1_-Arg extracts, an increase in total immobility time with mice was 1.21-fold (*p* < 0.05), 1.20-fold (*p* < 0.05), 1.71-fold (*p* < 0.05) and 1.29-fold (*p* < 0.05), respectively (compared to intact mice). The values for a total immobility time, however, were lower than those observed with a comparison group. Therefore, the present Tail Suspension Test results (Table 5) indicate that the V_1_, V_1_-Phe and V_1_-Lys extracts present antidepressant activity in mice. The V_2_, V_1_-Gly and V_1_-Arg extracts did not show antidepressant effects, but these three extracts demonstrated a mild sedative activity. However, the sedative activity in mice was significantly lower than that found in the mice treated with “Sedaphyton”. The V_1_-Val extract, however, exhibited a sedative activity in mice comparable to “Sedaphyton”. In conclusion, the V_1_ extract showed strong anxiolytic and antidepressant effects in mice, while the V_1_-Val extract consistently exhibited pronounced sedative activity across all studies.

### 2.3. Novel 3D-Printed Oral Dosage Forms for the V. officinalis Extract

**Figure 1:**
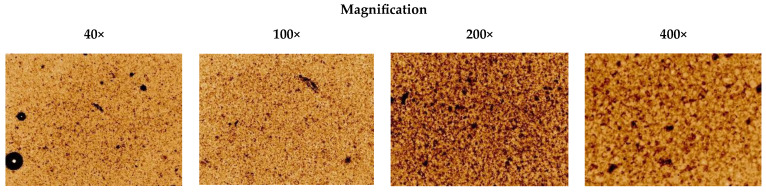
Optical light microscopy images of the PEO gels loaded with the *V. officinalis* extract (1.0 g in 10 g of the gel). Magnification 40×, 100×, 200× and 400×.

**Figure 2:**
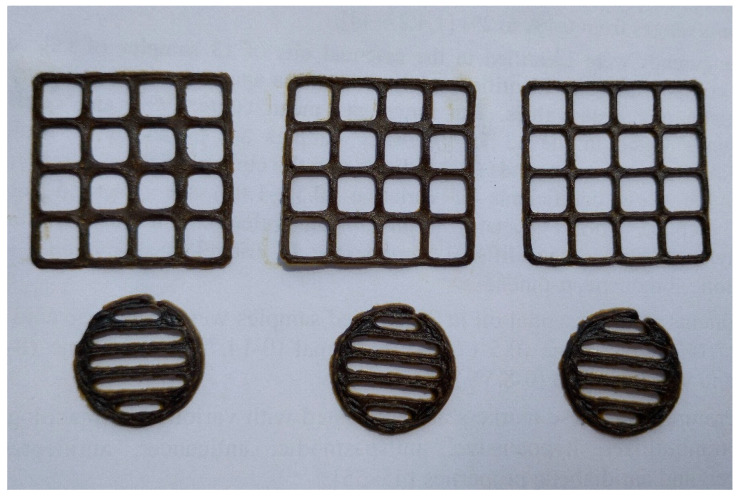
Photographs of the 3D-printed scaffolds (lattices and discs) loaded with the *V. officinalis* extract.

The PEO printing gel loaded with the dry *V. officinalis* extract (V_1_) appeared as a greenish-brown viscous semi-solid with a distinct odour. The gel was quite homogeneous, as proven by a microscopic analysis (Figure 1). The viscosity of gel, assessed at a rotational speed of 0.03 RPM and a shear rate of 0.060 1/s (22 ± 2 °C), was 209,900 ± 26,540 cP. The standard square- and round-shaped 3D-printed scaffolds were prepared by using an aqueous PEO gel loaded with the *V. officinalis* extract for SSE 3D printing (Figure 2). The printing quality of the preparations was studied by measuring the surface area (396.75 ± 60.08 mm^2^), S_practical/_S_theoretical_ ratio (1.22) and the average mass of the printed scaffolds (lattices 214.4 ± 21.4 mg and discs 175.0 ± 1.0 mg).

## 3. Discussion

The main identified phenolic compounds in the dry *V. officinalis* extracts were isovanillin, quercetin and protocatechuic acid. According to the literature, gallic and ferulic acids, quercetin and rutin have effects on the central nervous system (CNS) [44,45,46,47,48]. Gallic acid presents neuroprotective effects by reducing oxidative stress and inflammation in the brain. Gallic acid may also improve cognitive function and protect neurons from degeneration [50]. Ferulic acid has antioxidant and anti-inflammatory properties, and it could play a role in the prevention of neurodegenerative disorders like Alzheimer’s disease [51]. Rutin and quercetin are flavonoids that may enhance cerebral circulation, reduce inflammation and promote neuronal protection [52,53,54]. *p*-Coumaric acid modulates hypothalamic AMPK activity and enhances leptin signalling, thus improving glucose homeostasis and reducing food intake [55]. Protocatechuic acid has been reported to enhance cognitive function, reduce neuronal apoptosis and improve synaptic plasticity by regulating dopamine turnover, by inhibiting oxidative stress and inflammation and by modulating BDNF and MAPK signalling [56,57]. Syringic acid acts as a neuroprotective agent in the models of Alzheimer’s disease, Parkinson’s disease and neurotrauma [58]. With isovanillin, however, there is only limited direct evidence on CNS effects. Structurally, it is related to vanillin, which has mild neuroactive properties. To date, the effects of isovanillin on the CNS are not well-established in the state-of-the-art literature.

Among the 17 identified amino acids (Table 1), the following eight are essential: leucine, valine, phenylalanine, lysine, histidine, isoleucine, threonine and tryptophan. The dominant amino acids identified were asparagine, glutamine, α-alanine, arginine, threonine and valine. Leucine, valine, phenylalanine and lysine have effects on the CNS. Leucine is involved in neurotransmitter synthesis and brain metabolism regulation. It activates the mTOR signalling pathway, thus influencing neuron growth and survival [59]. Valine is essential for neurotransmitter synthesis and supporting cognitive function. The recent studies confirm its role in brain metabolism and show potential impact on neurodegenerative diseases [60]. Phenylalanine is a precursor to dopamine, norepinephrine and adrenaline, and it influences mood, motivation and cognitive function [61]. Lysine was shown to affect serotonin levels and thus help to reduce stress and anxiety [60]. These amino acids play vital roles in CNS function, and their effects are actively studied in the context of neurodegenerative diseases and cognitive disorders.

The pharmacological studies here revealed that the verbena extracts (unmodified and modified with amino acids) exhibit diverse biological activity (i.e., the anxiolytic, antidepressant, locomotor and sedative effects). The non-modified extracts may influence the GABAergic, serotonergic and dopaminergic systems, which explains their anxiolytic and antidepressant effects [62,63].

It is evident that the amino acid-modified extracts enhance the inhibitory processes in the CNS. This is primarily due to valine, which possesses neurotrophic and inhibitory properties affecting the CNS. Valine can reinforce inhibitory processes, either indirectly through metabolism or directly via GABA receptor interactions. As a result, these compounds exhibit sedative effects, manifested as reduced locomotor activity, prolonged time spent in dark compartments and fewer transitions into illuminated areas. Moreover, valine improves drug absorption and facilitates passage through the blood–brain barrier (BBB) via LAT1 (Large Amino Acid Transporter 1), thus enabling the release of active compounds in the brain. The sedative effect observed in mice following administration of V_1_-Val may be attributed to the presence of specific bioactive compounds within *V. officinalis* extract profile. Several studies have demonstrated that flavonoids exhibit central nervous system depressant activity. Flavonoid glycosides such as rutin and quercetin derivatives have demonstrated anxiolytic and sedative properties through interactions with GABAergic pathways and other neuroreceptors [64]. V_1_-Val contains similar constituents; its pharmacological profile may explain the behavioural outcomes observed in the study. A similar trend was observed in the study of amino acid modifications of motherwort tincture, specifically the complex with valine, which showed the best results and proved to be the most promising substance with neurotropic activity [39]. This suggests that valine also exhibits a synergistic effect with flavonoids. However, valine may also inhibit serotonin synthesis through the competitive transport of tryptophan via LAT1, which in turn enhances neurotropic activity and alters neurotransmitter balance, thus leading to reduced serotonin levels [65,66,67,68].

Arginine, as a modifying amino acid, can significantly affect drug bioavailability and BBB penetration by improving the solubility and absorption of the drug substance. The verbena extract with arginine utilises CAT-1 (Cationic Amino Acid Transporter 1) in the endothelial cells of the BBB to facilitate brain entry. Since CAT-1 regulates brain arginine levels, which serves as a precursor for nitric oxide (NO), it influences neurotransmission, neuroinflammation and cerebral blood flow. NO can modulate neuronal activity, thus leading to sedation. The mice receiving the V_1_-Arg avoided open areas and exhibited reduced activity, thus indicating sedative effects. This explains the weaker anxiolytic action and stronger sedative effect of the arginine-modified extract [69,70,71].

We propose that by modifying the verbena extract with specific amino acids, one can alter its pharmacological profile and shift its activity from anxiolytic to sedative effects, depending on the neurochemical pathways activated or inhibited. Previously, various animal studies have shown an increase in the potency of various pharmacological effects of extracts prepared from plants such as *Arctostaphylos uva-ursi* L. [34], *Matricaria chamomilla* L. [35], *Oxycoccus marocarpus* (Hill) A.Gray [36], *Solidago canadensis* L. [72], etc. The effect on a particular effect depends on the specific amino acid, which must be determined as a result of the experiment.

The aqueous PEO-based gel containing 1.0 g of the *V. officinalis* extract (V_1_) per 10 mL of gel was successfully used for SSE 3D printing. The 3D-printed lattices and round disc-shaped preparations exhibited consistent shape and dimensions (Figure 2). The applicability and compatibility of PEO as a carrier polymer with *V. officinalis* extract in the gel-based printing inks were verified. In our previous studies, we used surface-active agents to improve the 3D-printed formulations of German chamomile [35], *Solidago canadensis* [72] and eucalyptus extracts [43,73]. In these studies, it was shown in the pilot in-vitro disintegration tests that the SSE 3D-printed PEO discs loaded with the plant-based extract lost their structural integrity and fully disintegrated within 20–25 min in purified water at room temperature (22 ± 2 °C). Since all samples were completely disintegrated within 30 min, such 3D-printed preparations showed potential as immediate-release oral delivery systems [74,75].

Medicinal products derived from plants, including *V. officinalis* extract, are generally associated with high safety and tolerability. Consequently, herbal tinctures and extracts are widely incorporated into treatment regimens to enhance therapeutic effectiveness. Despite their benefits, galenic formulations such as teas, decoctions, tinctures and liquid extracts encounter limitations related to inconsistent standardisation and reduced patient compliance. One innovative approach to address these challenges involves applying pharmaceutical 3D-printing technologies to develop novel oral dosage forms. This strategy may significantly improve both the performance and acceptability of phytomedicines. Successfully engineering 3D-printed dosage forms containing plant-based substances, including *V. officinalis* extracts, could contribute to more rational and individualised application of herbal therapies in modern pharmaceutical and clinical settings.

For further development, it would be important to establish the standardisation techniques for these 3D-printed herbal dosage forms. Moreover, both preclinical and clinical trials should be undertaken to verify the safety and efficacy of the preparations. The pharmaceutical, chemical, and microbiological quality of these preparations should be validated through the successful completion of all development stages. Currently, the regulatory framework and market authorisation guidelines for 3D-printed medicinal products remain unclear and underdeveloped. This, in turn, creates an obstacle to the adoption of *V. officinalis* extract-based printed formulations for wider use. These future aspirations are supported by a significant advantage of 3D-printed preparations of *V. officinalis*: the application of this technology (for example, in a war situation or other emergency) is mobile and ensures individuality in the dosage of the extract for a specific patient.

## 4. Materials and Methods

### 4.1. Materials

The *V. officinalis* herb was collected in 2023 from the Kubja Ürditalu herb farm, Estonia. To prepare the dry extracts, 300 g of *V. officinalis* herb was macerated in 1500 mL of 70% aqueous ethanol at room temperature for 24 h. The liquid extract was then separated, and the extraction process was repeated once more using another 1500 mL of 70% ethanol solution. The two liquid extracts were combined, settled for two days and filtered. From the combined extract, five 200 mL portions were taken, with each portion receiving a different amino acid: 0.3705 g glycine (OstroVit, Zambrov, Poland), 0.8153 g phenylalanine (OstroVit, Zambrov, Poland), 0.7212 g L-lysine (FITS, Tallinn, Estonia), 0.5780 g valine (Acros Organics, Geel, Belgium) and 0.8596 g L-arginine (FITS, Tallinn, Estonia). Amino acids were added in a threefold equimolar ratio relative to the phenolic content, calculated based on gallic acid equivalence. Since gallic acid contains three free hydroxyl groups, we theoretically aimed to maximise conjugation at these functional sites. The amino acid solutions and the remaining liquid extract were infused for 24 h, followed by evaporation using a Buchi B-300 rotary vacuum evaporator (Buchi AG, Flawil, Switzerland) to obtain soft extracts. These were then freeze-dried (lyophilised) using a SCANVAC COOLSAFE 55-4 Pro freeze dryer (LaboGene ApS, Lillerød, Denmark). The final dry extracts were designated as V_1_, V_1_-Gly, V_1_-Phe, V_1_-Lys, V_1_-Val and V_1_-Arg.

For preparing the aqueous extract V_2_, 100.0 g of dried *V. officinalis* herb was used as an infusion with 1250.0 mL of water, heating up to 100 °C within 15 min and macerated within 24 h. After that, the aqueous extract was separated from the plant material through paper filtration and converted into a dry extract (V_2_) through lyophilisation by using a SCANVAC COOLSAFE 55-4 Pro freeze dryer (LaboGene ApS, Lillerød, Denmark).

### 4.2. Assay of Main Phytochemicals by Spectrophotometry

The determination of key phenolic compounds (hydroxycinnamic acids, flavonoids and total phenolic substances) in the dry extract of *V. officinalis* herb and its amino acid preparations was performed using a Shimadzu UV-1800 spectrophotometer (Shimadzu Corporation, Kyoto, Japan) according to the European Pharmacopoeia protocols. The quantification of hydroxycinnamic acids was conducted using a reaction with sodium molybdate and sodium nitrite. Chlorogenic acid was used as an equivalence standard [16,72]. The flavonoid content was determined based on a reaction with aluminium chloride, and the absorbance was measured at the wavelength of 417 nm [16,72]. Rutin was used as a reference compound. Total phenolic compounds were analysed at the wavelength of 270 nm by using gallic acid as the standard [76,77]. To ensure statistical accuracy, each experiment was conducted in triplicate.

### 4.3. Analysis of Phenolic Compounds by LC-MS/MS

The qualitative and quantitative assessment of phenolic compounds in the *V. officinalis* extracts was carried out with a triple quadrupole LC-MS/MS system using an Agilent 1290 Infinity chromatography system (Agilent Technologies, Santa Clara, CA, USA). The temperature of the column (Acquity UPLC BEH C18 column (2.1 × 100 mm, 1.7 µm) from Waters Corp., Milford, MA, USA) was kept at 45 °C. The mobile phase flow rate was 0.3 mL/min with solvent A (0.1% aqueous formic acid) and solvent B (methanol). The gradient-based elution was performed as follows: (1) from 0 to 4 min, the concentration of solvent B was maintained at 5%; (2) from 4 to 7.5 min, the concentration of solvent B was increased to 21% and kept at 21% till 8 min; (3) from 8 to 11 min, the concentration of solvent B was increased to 25% and then kept 25% till 13 min; (4) from 13 to 15 min, the concentration of solvent B was increased to 95%; and this was followed by (5) a column wash step with 95% solvent B from 15 to 20 min and re-equilibration to the initial conditions (5% solvent B) from 21 to 30 min. An Agilent 6495 triple quadrupole tandem mass spectrometer (Agilent Technologies, Santa Clara, CA, USA) was used for detection. All phenolic compounds were detected using heated electrospray ionisation (ESI, Agilent JetStream, Agilent Technologies, Santa Clara, CA, USA, in negative ionisation mode. The data was recorded using a dynamic MRM scan type. The following ESI–MS parameters were used: capillary voltage −3 kV, the temperature of sheath gas (N_2_) 350 °C and flow rate 12 L/min. The identification of phenolics was carried out by comparing the retention times and MS/MS spectral data with standards. Samples and standards were prepared in a solvent with 20% methanol and 80% 0.005 M aqueous HCl [78,79].

### 4.4. Assay of Amino Acids by LC-MS/MS

The amino acids assay in the *V. officinalis* extracts was carried out using an Agilent 1290 Infinity LC system equipped with an Agilent 6460 triple quadrupole mass spectrometer. An Agilent (Agilent Technologies, Santa Clara, CA, USA) Eclipse plus C18 column (50 mm × 2.1 mm, 1.8 µm) was used at 45 °C. The mobile phase consisted of 0.1% aqueous formic acid (eluent A) and acetonitrile (eluent B). The flow rate was 0.4 mL/min. The gradient-based elution was started with a 10% eluent B from 0 to 1 min. From 1 to 1.5 min, the concentration of eluent B was increased to 15%, and it was kept at 15% till 4.5 min. From 4.5 to 6.5 min, the concentration of eluent B was increased to 35%, and it was kept at 35% till 12 min. From 12 to 13 min, the concentration of eluent B was increased to 100% and maintained at 100% till 16 min. From 16 to 17 min, the gradient was returned to the initial conditions and kept till 23 min for column equilibration. The mass spectrometer was operated in positive electrospray ionisation (ESI, Agilent JetStream, Santa Clara, CA, USA) mode. The data was recorded using a dynamic MRM scan type. The following ESI–MS parameters were used: capillary voltage +3.5 kV, sheath gas flow 12 L/min and temperature 400 °C. The identification of amino acids was carried out by comparing the retention times and MS/MS spectral data with standards. Amino acids were analysed following the derivatisation with dethylethoxymethylene malonate (DEEMM) as follows: to 100 µL of sample (or standard) solution, 7 µL of DEEM, 518 µL of 0.1 M HCl in 30% methanol and 875 µL of borate buffer 0.75 M (pH 9.8) were added. Solutions were mixed for 1 min and injected into the LC-MS/MS [80,81].

### 4.5. Pharmacological Research

The study of neurotropic activity of the *V. officinalis* extracts was conducted in accordance with the methodological guidelines of the State Expert Centre of the Ministry of Health of Ukraine [44] at the clinical–biological experimental base of Ivano-Frankivsk National Medical University (IFNMU).

All animal experiments were conducted in strict accordance with the National General Ethical Principles of Animal Research (Ukraine, 2001). These Ethical Principles are aligned with the European Convention for the Protection of Vertebrate Animals Used for Experimental and Other Scientific Purposes (Strasbourg, 1986) [82,83,84]. Additionally, the study adhered to ethical, moral and legal standards aimed at ensuring the humane treatment of experimental animals in scientific and educational contexts, as outlined in Protocol No. 151/25 of the Ethics Committee of IFNMU, approved on 10 April 2025.

The study of neurotropic activity of the *V. officinalis* extracts was conducted with white outbred sexually mature mice of both sexes (8–12 weeks of age, weighing 20–24 g) that were bred and housed in the vivarium of IFNMU. The animals were standardised based on physiological and biochemical parameters and maintained in accordance with sanitary, hygienic norms. The animals received a standard diet while ensuring humane treatment.

The mice were housed under standard sanitary conditions at 20–24 °C, relative humidity of 50–55%, and a natural light cycle (“day-night”). They were kept in plastic cages with a balanced diet in compliance with current regulations. The study was conducted following the guidelines of “Preclinical Studies of Medicinal Products” [44].

To evaluate the neurotropic effects of the verbena extracts, the following behavioural pharmacology tests were used: Open Field Test, Elevated Plus Maze and Black-and-White Box Test [44,45,46].

A Tail Suspension Test was used to investigate antidepressant activity. The present test models despair-based behaviour [46,47,48]. Each test was conducted on a separate day.

The animals were divided into nine groups (six mice per group): (1) control animals (control group); (2) comparison group—the mice receiving “Sedaphyton” (Fitopharm, Ternopil, Ukraine); (3) the mice receiving verbena extract (solvent: 70% ethanol) (V_1_); (4) the mice receiving verbena extract (solvent: purified water) (V_2_); (5) the mice receiving verbena extract with glycine (V_1_-Gly); (6) the mice receiving verbena extract with phenylalanine (V_1_-Phe); (7) the mice receiving verbena extract with lysine (V_1_-Lys); (8) the mice receiving verbena extract with valine (V_1_-Val); and (9) the mice receiving verbena extract with arginine (V_1_-Arg). The tested verbena extracts and the reference drug were administered to the animals as a single oral dose using a specialised gavage tube, after being dissolved in water, at a dosage of 50 mg/kg (based on a volume of 0.1 mL per 10 g of body weight).

### 4.6. Three-Dimensional (3D) Printing of V. officinalis Extracts

A polyethylene oxide (PEO) gel was prepared for SSE 3D printing by adding PEO (MW ~900,000, Sigma-Aldrich, St. Louis, MO, USA) in purified water at a 12% (*w*/*w*) concentration. The preparation involved dispersing 1.2 g of PEO in 10 mL of purified water and allowing it to hydrate at room temperature for 13–15 h [43,85]. Tween 80 (Laborat GMBH, Berlin, Germany) was mixed in the gel to improve gel stability, to maintain homogeneity and to enhance the release of *V. officinalis* extract from the printed scaffolds [43,73]. The final printing gel formulation consisted of *V. officinalis* extract 1.0 g and Tween 80 0.5 g as a surfactant. The gel viscosity was measured at 22 ± 2 °C using a Physica MCR 101 rheometer (Anton Paar, Graz, Austria). For 3D printing, a bench-top Hyrel 3D printer (System 30 M, Hyrel 3D, Norcross, GA, USA) was used. The printing operation was controlled via Repetrel software, Rev3.083_K (Hyrel 3D, Norcross, GA, USA). The SSE 3D-printing head speed was set at 0.5 mm/s. The SSE 3D printer was equipped with a blunt needle (Gauge 21G), and no heating was applied to the syringe or printing platform. Two types of 3D-printed constructs were prepared and evaluated: (1) a square-shaped 4 × 4 grid lattice (30 × 30 × 0.5 mm) and (2) a round scaffold with a diameter of 20 mm.

The 3D models were generated using Autodesk 3ds Max Design 2017 (Autodesk Inc., San Francisco, CA, USA) and FreeCAD (version 0.19, released in 2021) [86]. The lattices consisted of six printed layers, while the round scaffolds had five layers. The printed preparations were left to air dry on the printing platform at a room temperature (22 ± 2 °C) before removal. To evaluate 3D printability, the weight and surface area of the printed preparations were measured. The theoretical surface area of the lattice structure was 324 mm^2^, and this value was compared with the actual surface area of the experimentally printed lattices [43,85]. The images of the printed preparations were processed using ImageJ software (National Institute of Health, Bethesda, MD, USA, version 1.51k). The weight of the preparations was determined with an analytical balance (Scaltec SBC 33, Scaltec, Göttingen, Germany).

### 4.7. Statistical Analysis

All experimental data were analysed using basic statistical methods, including the calculation of arithmetic mean values and standard deviations. For statistical validation, Student’s *t*-test was employed with a significance level of *p* ≤ 0.05. Additionally, to assess differences among multiple groups in behavioural tests, we have incorporated appropriate multiple comparison procedures, such as one-way ANOVA followed by post hoc analysis (e.g., Tukey’s test), where applicable. All computations were conducted using Microsoft Excel 2007 (Microsoft Corporation, Redmond, WA, USA), in accordance with the guidelines outlined in the State Pharmacopoeia of Ukraine [87,88].

## 5. Conclusions

The chemical profile and neurotropic effects of *V. officinalis* dry extract and its amino acid-based preparations were investigated. A total of eight main phenolic compounds and 17 amino acids were found in the *V. officinalis* dry extracts. The dry aqueous–ethanolic extract (extractant 70% ethanol) demonstrated strong anxiolytic and antidepressant effects, while the dry extract modified with valine and arginine consistently exhibited pronounced sedative activity across all studies. The novel aqueous gel formulation loaded with the *V. officinalis* dry extract was successfully designed and used for SSE 3D-printing applications. The SSE 3D printing enables the fabrication of new oral dosage forms for *V. officinalis* dry extracts.

## Figures and Tables

**Table 1:** Content of identified phenolic compounds and amino acids in the *V. officinalis* extracts.

Compound	$\mathrm{Content}\;\mathrm{in}\;\mathrm{the}\;\mathrm{Dry}\;\mathrm{Extract},\;\mathrm{mg}/\mathrm{kg},\;\bar{x}\pm \Delta \bar{x},\;\mathrm{(n = 3)}$						
	V_1_ (Extractant 70% Ethanol)	V_2_ (Extractant Water)	V_1_-Gly	V_1_-Phe	V_1_-Lys	V_1_-Val	V_1_-Arg
Phenolic compounds							
*p*-Coumaric acid	14 ± 3	25 ± 5	8 ± 1	13 ± 1	15 ± 2	15 ± 1	12 ± 5
Quercetin	285 ± 10	137 ± 6	137 ± 8	231 ± 11	240 ± 3	217 ± 5	208 ± 8
Gallic acid	11 ± 3	102 ± 14	13 ± 1	16 ± 2	13 ± 1	13 ± 1	11 ± 1
Protocatechuic acid	79 ± 11	116 ± 27	91 ± 14	168 ± 12	78 ± 11	82 ± 11	76 ± 8
Syringic acid	54 ± 3	101 ± 6	51 ± 7	62 ± 15	70 ± 8	66 ± 11	60 ± 7
Isovanillin	5405 ± 1432	6414 ± 1514	983 ± 281	5435 ± 847	824 ± 70	3401 ± 533	2519 ± 354
Ferulic acid	33 ± 1	53 ± 5	21 ± 3	36 ± 3	35 ± 1	31 ± 2	27 ± 2
Rutin	14 ± 2	7 ± 1	6 ± 0	16 ± 1	14 ± 0	11 ± 1	10 ± 1
Amino acids							
Histidine	27 ± 4	52 ± 4	17 ± 3	33 ± 7	31 ± 9	27 ± 7	23 ± 6
Arginine	187 ± 7	289 ± 8	154 ± 6	216 ± 14	195 ± 9	175 ± 13	134,100 ± 2600
Asparagine	1253 ± 56	2238 ± 77	1114 ± 51	1533 ± 24	1265 ± 93	1141 ± 41	1196 ± 46
Glutamine	4995 ± 533	666 ± 37	3958 ± 124	5839 ± 27	5574 ± 1219	4501 ± 324	4612 ± 333
Serine	329 ± 22	421 ± 14	317 ± 10	411 ± 10	356 ± 34	326 ± 11	310 ± 9
Aspartic acid	345 ± 36	359 ± 36	295 ± 6	331 ± 29	306 ± 50	292 ± 22	286 ± 16
Glycine	46 ± 5	90 ± 5	58,500 ± 1900	489 ± 8	57 ± 8	72 ± 4	54 ± 2
Threonine	121 ± 3	181 ± 3	128 ± 4	172 ± 1	132 ± 8	120 ± 3	118 ± 2
β-Alanine	29 ± 6	43 ± 7	34 ± 2	50 ± 2	33 ± 7	28 ± 5	37 ± 3
α-Alanine	721 ± 30	915 ± 45	763 ± 9	919 ± 12	789 ± 65	950 ± 24	722 ± 13
Tyrosine	71 ± 4	113 ± 1	70 ± 3	167 ± 4	73 ± 1	114 ± 4	75 ± 7
Valine	97 ± 22	305 ± 47	196 ± 21	333 ± 50	135 ± 32	85,900 ± 3000	102 ± 32
Tryptophan	56 ± 9	74 ± 9	50 ± 4	78 ± 9	56 ± 6	53 ± 6	46 ± 8
Phenylalanine	48 ± 11	106 ± 7	93 ± 6	183,500 ± 5600	57 ± 11	123 ± 8	55 ± 8
Isoleucine	93 ± 28	182 ± 26	124 ± 9	379 ± 28	94 ± 20	249 ± 23	108 ± 25
Leucine	73 ± 6	182 ± 4	97 ± 4	999 ± 34	78 ± 3	814 ± 40	86 ± 6
Lysine	26 ± 12	67 ± 13	27 ± 3	50 ± 7	68,000 ± 4200	53 ± 10	29 ± 8
Spectrophotometry, %							
Hydroxycinnamic acids (chlorogenic acid equivalents, spectrophotometry), %	5.79 ± 0.39	3.95 ± 0.31	5.51 ± 0.73	5.15 ± 0.47	4.46 ± 0.43	4.34 ± 0.67	3.78 ± 0.75
Flavonoids (rutin equivalents, spectrophotometry), %	2.91 ± 0.12	2.00 ± 0.09	2.48 ± 0.05	2.39 ± 0.06	2.27 ± 0.06	2.24 ± 0.08	2.07 ± 0.04
Total phenolic compounds (gallic acid equivalents, spectrophotometry), %	7.77 ± 0.28	5.75 ± 0.25	6.76 ± 0.38	6.26 ± 0.10	5.93 ± 0.18	5.85 ± 0.18	5.56 ± 0.16

Notes: V_1_—the dry *V. officinalis* extract, obtained with 70% aqueous ethanol and its amino acid preparations with glycine (V_1_-Gly), phenylalanine (V_1_-Phe), lysine (V_1_-Lys), valine (V_1_-Val) and arginine (V_1_-Arg); V_2_—the dry *V. officinalis* extract, obtained with water.

**Table 2:** Results of the study of the neurotropic activity of the *V. officinalis* extracts in the Open Field Test.

Investigated Indicator	Control Animals	Comparison Group (“Sedaphyton”)	V_1_ (Extractant 70% Ethanol)	V_2_ (Extractant Water)	V_1_-Gly	V_1_-Phe	V_1_-Lys	V_1_-Val	V_1_-Arg
Number of crossed squares	75.33 ± 5.68	73.17 ± 5.85	79.50 ± 4.55	94.17 ± 6.64 */**	80.50 ± 8.24	83.50 ± 3.77	81.50 ± 5.96	50.33 ± 5.27 */**	67.83 ± 7.46
Number of vertical stands	10.50 ± 1.77	7.83 ± 0.91	12.17 ± 1.25 **	11.17 ± 1.05 **	7.33 ± 0.67 *	10.67 ± 1.02 **	9.00 ± 0.58	6.67 ± 1.09 *	4.33 ± 0.49 */**
Number of hole explorations	14.17 ± 1.08	2.83 ± 0.48 *	12.50 ± 0.76 **	12.17 ± 0.98 **	11.00 ± 1.39 **	8.17 ± 0.48 */**	9.83 ± 0.95 */**	2.17 ± 0.60 *	3.17 ± 0.60 *
Conditional units of exploratory research activity	3	1.91	3.10	3.17	2.54	2.70	2.58	1.45	1.53
Boluses	1.33 ± 0.33	1.50 ± 0.81	1.50 ± 0.22	2.33 ± 0.42	2.67 ± 0.56	1.33 ± 0.21	1.00 ± 0.26	1.67 ± 0.21	1.17 ± 0.60
Grooming	0.67 ± 0.21	0.33 ± 0.33	1.17 ± 0.31	0.67 ± 0.33	0.67 ± 0.21	0.83 ± 0.17	1.00 ± 0.26	0.5 ± 0.22	0.33 ± 0.21
Conditional units of emotional reaction indicators	2	1.62	2.87	2.75	3	2.25	2.25	2	1.37
Conditional units of all activities	5	3.54	5.97	5.92	5.54	4.95	4.83	3.45	2.91

Note: *—significance of deviations relative to intact animal data (*p* < 0.05); **—significance of deviations relative to comparison group data (*p* < 0.05).

**Table 3:** Behaviour of animals in the Elevated Plus Maze test after the administration of the *V. officinalis* extracts.

Group of Animals	Time Spent in the Open Arm of the Maze, s	Time Spent in the Closed Arm of the Maze, s	Number of Peeks from the Closed Arm	Number of Crossings over the Central Platform	Number of Downward Glances from the Ends of Open Arms
Control animals	24.17 ± 3.95	273.17 ± 5.40	10.50 ± 1.80	3.33 ± 1.2	4.67 ± 0.61
Comparison group (“Sedaphyton”)	18.50 ± 1.84	279.00 ± 8.54	3.33 ± 0.42 *	1.00 ± 0.37 *	3.00 ± 0.63 *
V_1_ (extractant 70% ethanol)	54.67 ± 2.80 */**	235.67 ± 5.52 */**	8.50 ± 0.43 **	8.50 ± 1.06 */**	10.17 ± 1.05 */**
V_2_ (extractant water)	24.00 ± 2.53	270.17 ± 3.84	10.83 ± 1.35 **	6.33 ± 0.92 */**	5.33 ± 0.42 **
V_1_-Gly	29.33 ± 2.75 **	262.50 ± 6.57	3.17 ± 0.40 *	3.83 ± 0.06 **	4.33 ± 0.42
V_1_-Phe	59.50 ± 4.21 */**	238.00 ± 5.85 */**	10.33 ± 0.49 **	8.50 ± 1.06 */**	11.33 ± 1.20 */**
V_1_-Lys	47.17 ± 3.32 */**	250.50 ± 8.50 */**	8.00 ± 0.58 */**	5.50 ± 1.52 **	11.00 ± 1.41 */**
V_1_-Val	9.50 ± 1.43 */**	287.17 ± 1.96 *	2.67 ± 0.42 */**	1.83 ± 0.48 *	1.00 ± 0.37 */**
V_1_-Arg	23.50 ± 2.01	275.00 ± 5.94	4.67 ± 1.02 */**	3.00 ± 0.68 **	3.83 ± 1.25

Note: *—significance of deviations relative to intact animal data (*p* < 0.05); **—significance of deviations relative to comparison group data (*p* < 0.05).

**Table 4:** Anxiolytic activity of the *V. officinalis* extracts in the “Black-and-White Box” test.

Group of Animals	Time Spent in the Dark Compartment, s	Number of Peeks from the Dark Compartment	Time Spent in the Illuminated Compartment, s	Number of Exits into the Illuminated Compartment	Average Time of a Single Stay in the Illuminated Compartment, s
Control animals	101.00 ± 4.93	5.83 ± 1.17	78.33 ± 4.89	4.83 ± 0.31	16.73 ± 1.87
Comparison group (“Sedaphyton”)	119.00 ± 5.16 *	2.17 ± 0.70 *	59.67 ± 4.67 *	3.33 ± 0.71 *	25.68 ± 9.18
V_1_ (extractant 70% ethanol)	24.67 ± 5.28 */**	2.00 ± 1.06 *	100.33 ± 5.28 */**	3.17 ± 0.91	47.98 ± 12.85 **
V_2_ (extractant water)	98.33 ± 6.82 **	7.00 ± 1.15 **	80.17 ± 6.84 **	5.83 ± 0.54	14.14 ± 1.48
V_1_-Gly	75.67 ± 6.58 */**	2.33 ± 0.61 *	103.00 ± 6.61 */**	7.33 ± 0.71 */**	14.60 ± 1.33
V_1_-Phe	73.67 ± 5.49 */**	2.17 ± 1.22	105.5 ± 5.60 */**	6.33 ± 0.61 **	17.43 ± 1.82
V_1_-Lys	97.67 ± 10.55	3.83 ± 1.11	82.00 ± 10.56	5.33 ± 0.42 **	15.60 ± 1.97
V_1_-Val	104.33 ± 9.27	3.17 ± 1.08	75.33 ± 9.28	4.83 ± 0.87	20.23 ± 6.57
V_1_-Arg	126.67 ± 6.80 *	2.67 ± 0.92	53.83 ± 7.06 *	5.50 ± 1.12	11.66 ± 2.66

Note: *—significance of deviations relative to intact animal data (*p* < 0.05); **—significance of deviations relative to comparison group data (*p* < 0.05).

**Table 5:** Antidepressant activity of the *V. officinalis* extracts in the Tail Suspension Test.

Group of Animals	Total Immobility Time, s
Control animals	103.67 ± 5.00
Comparison group (“Sedaphyton”)	190.67 ± 12.44 *
V_1_ (extractant 70% ethanol)	84.67 ± 5.38 */**
V_2_ (extractant water)	125.00 ± 3.01 */**
V_1_-Gly	124.83 ± 7.96 **
V_1_-Phe	97.50 ± 5.76 **
V_1_-Lys	99.67 ± 6.25 **
V_1_-Val	177.50 ± 8.11 *
V_1_-Arg	133.67 ± 7.29 */**

Note: *—significance of deviations relative to intact animal data (*p* < 0.05); **—significance of deviations relative to comparison group data (*p* < 0.05).

## Data Availability

The data supporting the results of this study can be obtained from the corresponding authors upon reasonable request.
